# “Salvage Microbiology”: Detection of Bacteria Directly from Clinical Specimens following Initiation of Antimicrobial Treatment

**DOI:** 10.1371/journal.pone.0066349

**Published:** 2013-06-25

**Authors:** John J. Farrell, Rangarajan Sampath, David J. Ecker, Robert A. Bonomo

**Affiliations:** 1 University of Illinois School of Medicine, Department of Medicine, Peoria, Illinois, United States of America; 2 Ibis Biosciences, an Abbott Company, Carlsbad, California, United States of America; 3 Departments of Medicine, Pharmacology and Molecular Microbiology, Case Western Reserve University, Cleveland, Ohio, United States of America; 4 Louis Stokes Cleveland Department of Veterans Affairs Medical Center, Cleveland, Ohio, United States of America; University of Houston, United States of America

## Abstract

**Background:**

PCR coupled with electrospray ionization mass spectrometry (ESI-MS) is a diagnostic approach that has demonstrated the capacity to detect pathogenic organisms from culture negative clinical samples after antibiotic treatment has been initiated. [1] We describe the application of PCR/ESI-MS for detection of bacteria in original patient specimens that were obtained after administration of antibiotic treatment in an open investigation analysis.

**Methods:**

We prospectively identified cases of suspected bacterial infection in which cultures were not obtained until after the initiation of antimicrobial treatment. PCR/ESI-MS was performed on 76 clinical specimens that were submitted for conventional microbiology testing from 47 patients receiving antimicrobial treatment.

**Findings:**

In our series, 72% (55/76) of cultures obtained following initiation of antimicrobial treatment were non-diagnostic (45 negative cultures; and 10 respiratory specimens with normal flora (5), yeast (4), or coagulase-negative staphylococcus (1)). PCR/ESR-MS detected organisms in 83% (39/47) of cases and 76% (58/76) of the specimens. Bacterial pathogens were detected by PCR/ESI-MS in 60% (27/45) of the specimens in which cultures were negative. Notably, in two cases of relapse of prosthetic knee infections in patients on chronic suppressive antibiotics, the previous organism was not recovered in tissue cultures taken during extraction of the infected knee prostheses, but was detected by PCR/ESI-MS.

**Conclusion:**

Molecular methods that rely on nucleic acid amplification may offer a unique advantage in the detection of pathogens collected after initiation of antimicrobial treatment and may provide an opportunity to target antimicrobial therapy and “salvage” both individual treatment regimens as well as, in select cases, institutional antimicrobial stewardship efforts.

## Introduction

Infections are a major cause of morbidity and mortality in hospitalized patients. Because identification of bacterial pathogens requires time for the organisms to grow, when infection is suspected empiric antimicrobial treatment is administered based upon an assessment of likely organisms [2]–[4]. Bacterial meningitis is a primary example of the importance of appropriate empiric therapy before culture results are known. Here, a delay in the initiation of antimicrobial therapy correlates with increased mortality in this disease [5]–[7], and antibiotics are routinely administered before cerebrospinal fluid (CSF) is obtained (the site likely to yield the pathogen). Other clinical scenarios such as febrile neutropenia, transplant associated infections, and severe sepsis also mandate that antibiotics are administered in a timely fashion and should not be delayed.

The sensitivity and timeliness of culture results are influenced by many factors, but in hospitalized patients previously administered and/or concurrent antimicrobial treatment is a commonly encountered confounding factor. When pathogens are not recovered in culture, the entire treatment course is likely to be “broad spectrum” including therapy for staphylococci, streptococci, as well as Gram negative and anaerobic bacteria. As a result, antibiotics are often used unnecessarily. Regrettably, a delay or failure in identification of pathogens impacts patient outcomes, exposes patients to the deleterious effects of extended courses of overly broad empirical antibiotics, and exacerbates the widespread problems of multidrug resistant organisms and *Clostridium difficile* associated disease.

The analysis of multilocus polymerase chain reaction (PCR) amplicons by electrospray ionization mass spectrometry (ESI/MS) on a platform in which PCR is coupled to ESI-MS is a technique that has demonstrated the capacity to detect microbes from both environmental samples and clinical patient specimens [8]–[10]. Detection does not require correct anticipation of the organisms in advance; the technology is designed to detect unknown and unculturable organisms, and is particularly useful when multiple microbes may be present.

We performed a prospective comparison of results from conventional microbiologic testing vs. PCR/ESI-MS in cases of suspected new onset, or recurrence of infection in patients where samples were obtained after at least one dose of antibiotic treatment. Our purpose is to demonstrate that PCR/ESI-MS may have value in the clinical microbiology laboratory when cultures do not yield a pathogen. Offering clinicians relevant, timely and specific information can have significant impact on the choice of therapy, clinical decision making, and antimicrobial stewardship.

## Methods

Approval was obtained from both the University of Illinois College of Medicine and St. Francis Medical Center Institutional Review Boards (IRBs) for PCR/ESI-MS testing of specimens that were submitted to the microbiology laboratory from inpatients at St. Francis Medical Center, Peoria, IL. To test if PCR/ESI-MS serves a potential role in the detection of microorganisms in specimens collected from patients following administration of antimicrobial treatment, we prospectively identified patients with suspected infection between February 10, 2011 and November 10, 2012. Patients whose specimens were collected after at least one dose of antibiotic were included in the study. Gram stains, conventional aerobic and anaerobic culture and PCR/ESI-MS were performed on all specimens. The PCR/ESI-MS test results were not available to the patients' treatment teams and did not influence treatment decisions.

In each case, specimens were collected as part of the routine care of the patient and submitted to clinical microbiology lab at St. Francis Medical Center (Peoria, IL) for testing. After the specimen was processed by laboratory personal and all the requested tests and cultures ordered by the treating physician had been prepared, the remaining specimen was placed in storage at 4°C for subsequent PCR/ESI-MS testing. Because specimens included in this study were collected in the course of the patients medical care for diagnostic purposes, and no specimens were collected explicitly for the purposes of this study, both IRBs waived the requirement for patient informed consent.

PCR/ESI-MS was performed on all specimens. Specimens were kept in refrigeration, not frozen, and shipped overnight in a cold pack to Ibis Biosciences (Carlsbad, CA). We followed the PCR/ESI-MS protocol previously described [11]. This PCR/ESI-MS assay is designed for detection of bacterial and *Candida* species, and is not capable of identifying invasive molds, dimorphic fungi, or viral pathogens. Consequently, immunocompromised patients, patients on chemotherapy for treatment of malignancy, or patients with HIV infection were excluded from the study. Compared to clinical samples, the assay performs with 98.7% and 96.6% concordance at the genus and species levels, respectively [11].

For reporting results, the level of detection (LOD) was calculated as genome equivalents per PCR reaction well. Results were reported for all detections with a Q score ≥0.90 in which the LOD was above threshold, and the internal isolation control was detected. Kappa (κ) was calculated using SAS software, version 9.1 (SAS Institute) to assess the agreement between culture and PCR/ESI-MS.

## Results

Conventional microbiology testing was performed on 76 specimens collected from 47 patients. Specimens included swabs, BACTEC blood culture bottles, fluid, and tissue samples that were submitted for culture from patients after initiation of antimicrobial treatment (Table 1). The results obtained from aerobic and anaerobic cultures were compared to results of PCR/ESI-MS testing (Table 2). PCR/ESI-MS detected probable pathogens in 20 cases in which standard microbial cultures were non-diagnostic. Results were in agreement for 38 specimens (49%); but 37% of the agreement (14 specimens) was attributed to specimens that were culture negative with no detection by PCR/ESI-MS. For patients with multiple specimens, only the culture positive specimen, when applicable, was considered for calculation of the Kappa statistic. Compared to agreement between culture and Gram stain, (κ = 0.643), agreement between culture and PCR/ESI-MS was poor (κ = 0.299).

**Table 1:** Patients, specimens, and antimicrobial treatment.

Patient	Age/gender/history	Diagnosis	Previous culture confirmed infection(s)	Specimens	Antimicrobial Treatment	DOT*
1	48 year-old woman	Right lung abscess & empyema	–	Pleural fluid	Azithromycin Ceftriaxone Vancomycin	5 11 10
2	26 year-old	Brain		CSF #1 (from LP)	Ceftriaxone Vancomycin	1 1
	man	abscess	–	CSF #2 (from EVD)	Cefepime Ceftriaxone Clindamy cin Metronidazole Vancomycin	3 10 2 7 13
3	58 year-old man	AV IE with AV ring abscess	MSSE AV IE	AV tissue Annulus tissue	Gentamicin Rifampin Vancomycin	10 47 47
4	50 year-old man	Brain abscess	–	Purulent brain abscess fluid	Ceftriaxone Metronidazole Vancomycin	2 2 2
5	51 year-old man	Sepsis	–	BAL fluid	Clindamycin Levofloxacin Piperacillin/tazo bactam Vancomycin	2 2 2 2
6	74 year-old woman	Right shoulder	–	Synovial fluid	Antibiotics started after shoulder aspiration	0
	S/P shoulder reconstruction	septic arthritis		Synovial tissue	Ceftriaxone Vancomycin	3 3
7	50 year-old man	Sepsis	*S. pneumoniae*	CSF	Acyclovir Meropenem Vancomycin	3 3 3
			bacteremia	Brain tissue	Acyclovir Ceftriaxone Meropenem Vancomycin	7 4 3 7
8	10 year-old male	CAP	–	BAL fluid	Azithromycin	5
9	16 year-old male	CAP	–	Pleural fluid	Azithromycin Ceftriaxone	4 3
10	64 year-old man	right TKA septic arthritis	Methicillin resistant coagulase negative *Staphylococcus*	Synovial tissue; posterior femoral tissue; and posterior tibial tissue	Cefazolin	1
11	50 year-old man	Right hip AVN	–	Synovial fluid	Cefazolin Ceftriaxone Vancomycin	1 1 1
12	72 year-old woman	Right cranial epidural abscess	–	Epidural tissue	Cefazolin Ceftriaxone Vancomycin	1 1 1
13	59 year-old	Sepsis and	*S. pneumoniae*	CSF #1	Piperacillin/tazo bactam Vancomycin	6 6
	woman	meningitis	bacteremia	CSF #2	Ceftriaxone Piperacillin/ tazobactam Vancomycin	14 6 6
14	59 year-old man	Left TKA	*Alpha-Strep*	Synovial fluid from left knee (before surgery)	Antibiotics started after left knee aspiration	0
		septic arthritis	cultured from left knee fluid	Retinacular tissue and synovial tissue from the OR	Cefazolin	1
15	75 year-old man on chronic suppressive antibiotics	Recurrent septic left TKA	MSSA infected left TKA	Synovial fluid; synovial tissue; and femoral membrane tissue	Cefazolin Cephalexin Rifampin	6 360 360
16	86 year-old woman	Right TKA septic arthritis	MRSA infected left TKA	Synovial fluid	Cephalexin Clindamycin Linezolid	14 14 14
17	70 year-old woman	Encephalitis	–	CSF	Ceftriaxone Vancomycin	2 2
18	78 year-old woman	Liver abscess	–	Purulent liver abscess fluid	Piperacillin/tazobactam	1
19	55 year-old man	CAP	MSSA bacteremia	BAL fluid	Cefazolin Vancomycin	2 2
20	38 year-old woman	Submental abscess	–	Swab from I&D in OR	Clindamycin Vancomycin	2 1
21	78 year-old man	Severe AS	Culture negative endocarditis	AV tissue	Cefazolin Ceftriaxone Vancomycin	1 28 28
22	49 year-old man	Infective Endocarditis	*Abiotrophia* *defectiva* bacteremia	AV tissue and MV tissue	Cefazolin Vancomycin	2 2
23	79 year-old man	Sepsis	*Vibio vulnificans* bacteremia	BACTEC^TM^ blood culture bottles (two sets)	Piperacillin/tazo bactam Vancomycin	2 2
24	69 year-old woman	Acute Respiratory failure		ET aspirate	Meropenem Piperacillin/tazo bactam Vancomycin	4 3 6
			–	RLL and LLL BAL fluid	Meropenem Piperacillin/tazo bactam Vancomycin	4 4 7
25	40 year-old diabetic man	VAP	*Streptococcus agalactiae* (initial sputum culture)	RLL and LLL BAL fluid	Aztreonam Clindamycin Mer openem Vancomycin	6 10 4 4
26	68 year-old woman	CAP and ARDS	*E. coli* UTI	RLL and LLL BAL fluid	Azithromycin Ceftriaxone Meropenem Vancomycin	6 5 3 5
27	75 year-old man	Necrotizing		Wound aspirate POD #3	Ampicillin/sulbactam Clindamycin Mero penem Vancomycin	3 2 1 4
	with chronic sacral decubitus ulcer	fasciitis	–	Wound swab POD #15	Ampicillin/sulbactam Clindamycin Daptomycin Meropenem Vancomycin	15 2 12 1 4
28	33 year-old man with post-op wound infection	left ankle pilon fracture S/P ORIF	–	Aerobic and anaerobic swabs from OR	Cefazolin Cephalexin	1 7
29	49 year-old quadraplegic man	Stage 4 pressure ulcer	–	Left hip tissue from I&D in OR	Cephalexin Piperacillin/ tazobactam	5 1
30	68 year-old woman	Hypoxemia and		ET aspirate	Meropenem Vancomycin	1 1
		hypercapnic respiratory failure	–	RLL and LLL BAL fluid	Levofloxacin Meropenem Vancomycin	2 1 1
31	45 year-old woman with recurrent lower extremity infections	right knee septic arthritis	–	Swab of right knee fluid taken in OR	Levofloxacin Linezold	6 6
32	15 year-old female	Neck abscess	–	Right neck abscess tissue excised in OR	Azithromycin Cefdinir Clindamycin (po) Clindamycin (IV)	5 20 7 2
33	91 year-old man with small bowel obstruction	RUL collapse	–	RUL BAL fluid	Cefepime Metronidazole Vancomycin	5 5 4
34	51 year-old man S/P right to left femoral arterial bypass graft	post-op left groin seroma	*S. lugdunensis* bacteremia	Seroma fluid and Arterial Graft Material from OR	Aztreonam Cefazolin Levofloxacin Rifampin Vancomycin	2 3 1 5 4
35	74 year-old diabetic man	RUE cellulitis	–	Right elbow fluid	TMP/SMX DS	5
36	37 year-old woman with CBD leak	choledocholithiasis	–	Fluid from Peri-biliary abscess	Levofloxacin Meropenem Vancomycin	3 5 5
37	74 year-old woman	Right TKA effusion	–	Right femoral and tibial canal tissue from OR	Clindamycin Minocycline Tigecycline	1 4 6
38	50 year-old woman with RUL Adenocarcinoma	Necrotizing pneumonia	MSSA VAP	Fluid from right chest cavity	Ampicillin Ceftriaxone Levofloxacin Piperacillin/tazobactam Meropenem TMP/SMX Vancomycin	5 4 1 7 5 5 5
39	25 year-old woman S/P left ankle ORIF	left ankle osteomyelitis	Anaerobic streptococci	left ankle abscess tissue collected in OR	Clindamycin	31
40	71 year-old woman	LLL CAP	–	ET aspirate and LLL BAL fluid	Azithromycin Ceftriaxone Levofloxacin Piperacillin/tazobactam Vancomycin	5 9 14 2 2
41	84 year-old diabetic man	RLE cellulitis and diabetic right foot infection	*Streptococcus agalactiae* (wound culture)	Right 5th metatarsal bone	Cefepime Piperacillin/tazobactam Vancomycin	1 5 5
42	77 year-old woman with end stage renal disease on hemodialysis	left hip pain.	–	left hip joint tissue	Vancomycin	1
43	61 year-old man with h/o type A aortic dissection S/P modified Bentall surgery	Mediastinal abscess	*Salmonella enterica* serogroup enteritidis bacteremia	Aortic graft material, and fluid and tissue around aortic graft excised in OR	Ceftriaxone Levofloxacin Piperacillin/tazobactam Vancomycin	14 18 4 1
44	55 year-old man S/P left quadriceps tendon repair	Left knee septic arthritis	MSSA cultured from left knee fluid	Left knee tissue from OR	Cephalexin Vancomycin	4 5
45	56 year-old man with h/o anoxic brain injury	respiratory distress	–	ET aspirate and RLL BAL fluid	Amoxacillin Levofloxacin Piperacillin/tazobactam Vancomycin	2 1 2 2
46	41 year-old man with hypercapnic respiratory failure	Drug overdose	–	ET aspirate and RLL BAL fluid	Ampicillin/sulbactam	3
47	77 year-old woman with lung cancer	HCAP	*Pseudomonas aeruginosa* pneumonia	ET aspirate	Amikacin Colistimethate Doripenem Piperacillin/tazobactam Vancomycin	10 8 5 14 14

* **DOT**– Days of therapy; **POD** – Post-operative Day; **S/P** – Status-post; **LP** – lumbar puncture; **I&D** – incision and drainage.

**MSSA** – Methicillin susceptible *Staphylococcus aureus* **; CBD** – Common bile duct; **TMP/SMX** – Trimethoprim-Sulfamethoxazole.

**RLE/LLE** – right/left lower extremity; **TKA**– Total Knee Arthroplasty; **ORIF**– Open Reduction and Internal Fixation.

**UTI** – Urinary tract infection; **AVN** – avascular necrosis; **IE** – infective endocarditis; **AV** – aortic valve; **MV** – mitral valve.

**CAP**–Community acquired pneumonia; **VAP**–Ventilator associated pneumonia; **HCAP**–Healthcare associated pneumonia.

**BAL** – bronchoalveolar lavage; **RLL/LLL** – right/left lower lobe; **ET** – endotracheal tube; **ARDS** – acute respiratory distress syndrome.

**Table 2:** Conventional microbiology versus PCR/ESI-MS test results.

	Respiratory and pulmonary specimens						
Patient	Specimen	Gram Stain	Culture results		PCR/ESI-MS		Level (GE/well)#
1	Pleural fluid	No segs No organisms	No growth		*Streptococcus pneumoniae/mitis* Group streptococci		75
5	BAL fluid	Few segs No organisms	Light growth of normal respiratory flora		*Streptococcus pneumoniae*		54
8	RLL BAL fluid	Many segs No organisms	No growth		*Streptococcus pneumoniae*		54
9	Left pleural fluid	Many segs No organisms	No growth		No detection		–
19	BAL fluid	Few segs	Sparse normal		*S. aureus* (*mec*A negative)		55
		No organisms	respiratory flora		*C. albicans*		125
	ET aspirate	Some segs No organisms	Rare Coagulase negative Staphylococcus		*S. pneumoniae C. albicans*		58 135
24	RLL BAL fluid	No segs No organisms	No growth		No detection		–
	LLL BAL fluid	No segs No organisms	No growth		No detection		–
25	RLL BAL fluid	Rare segs No organisms	*C. glabrata*		No detection		–
	LLL BAL fluid	Rare segs No organisms	*C. glabrata*		*C. glabrata*		1005
26	RLL BAL fluid	No segs No organisms	Rare *C. albicans*		*C. albicans*		140
	LLL BAL fluid	No segs No organisms	No growth		*C. albicans*		141
	ET aspirate	Some segs No organisms	Normal respiratory flora		*S. pneumoniae C. albicans*		58 135
30	RLL BAL fluid	Few segs No organisms	No growth		*S. vestibularis C. albicans*		98 116
	LLL BAL fluid	Rare segs No organisms	No growth		*S. pneumoniae C. albicans*		68 112
33	RUL BAL fluid	Some segs Rare budding yeast	Many *C. albicans*		*Bifidobacterium dentium C. glabrata*		39 126
38	Right chest pleural	Many segs	Many MSSA		*S. aureus* (*mec*A negative)		72
	fluid	Few budding yeast	Few *C. albicans*		*C. albicans*		119
		Many segs	Sparse		*Rothia mucilaginosa*		60
	ET aspirate	Rare GPC	Normal respiratory flora		*Staphylococcus epidermidis*		28
					*C. albicans*		899
40					*Rothia mucilaginosa*		30
	LLL BAL fluid	Rare segs No organisms	No growth		*S. epidermidis* (*mec*A positive)		12
					*C. albicans*		27
		Many segs			*Escherichia coli*		118
	ET aspirate	rare GNB	Many *E. coli*		*S. epidermidis* (*mec*A positive)		11
45		Many budding yeast			*Candida tropicalis*		138
	RLL BAL fluid	Many segs, rare GPC	Some E. coli		*Escherichia coli*		103
		Rare budding yeast	Many *C. tropicalis*		*Candida tropicalis*		138
		Some segs	Sparse		viridans/mitis Group streptococcus		95
	ET aspirate	No organisms	Normal respiratory flora		*Streptococcus* spp.		137
46					*C. albicans*		132
	RLL BAL fluid	No segs No organisms	No growth		viridans/mitis Group streptococcus *C. albicans*		64 61
		Many segs	Few		*Pseudomonas aeruginosa*		295
47	ET aspirate	Rare GNB	*Pseudomonas aeruginosa*		*Streptococcus* spp.		29
				*C. albicans*		25	

	Tissue, Fluid, and Orthopedic Specimens				
Patient	Specimen	Gram Stain	Culture results	PCR/ESI-MS	Level (GE/well)#
	Right shoulder	Many segs	No	*Propionibacterium acnes*	17
6	Synovial fluid	No organisms	growth	*Acinetobacter junii*	7
	Right shoulder tissue	No organisms	No growth	*Acinetobacter junii*	188
	Synovial tissue	Negative	No growth	*S. epidermidis* (*mec*A positive)	41
10	Posterior femoral tissue	Negative	No growth	*S. epidermidis* (*mec*A positive)	201
	Posterior tibial tissue	No segs No organisms	Methicillin resistant coagulase negative *Staphylococcus* (one colony in the anaerobic culture)	*S. epidermidis* (*mec*A positive)	113
11	Synovial fluid	Some segs No organisms	No growth	No detection	–
	Left knee fluid	Many segs. Rare GPC in pairs	Alpha-hemolytic streptococcus	*S. pneumoniae*/viridans/mitis group streptococci	139
14	Left knee retinaculum	Rare segs No organisms	No growth	*S. pneumoniae*/viridans/mitis group streptococci	135
	Left knee femoral synovial tissue	Few segs No organisms	No growth	*S. pneumoniae*/viridans/mitis group streptococci	134
	Left knee fluid	No organisms	No growth	*S. aureus* (*mec*A negative)	44
15	Left knee synovial tissue	Rare segs No organisms	No growth	Low level detection. Unable to identify organism.	–
	Left knee femoral membrane tissue	No segs. No organisms	No growth	*S. aureus* (*mec*A negative)	96
	Left knee	No segs	No	*S. aureus* (*mec*A positive)	34
16	tissue	Rare GPC	growth	*Acinetobacter junii*	99
	Tibial bone tissue	Few segs. No organisms	No growth	No detection	–
18	Fluid aspirated from liver abscess	Few segs	Many *Streptococcus intermedius*	*Streptococcus* spp.	40
		No organisms	Many *Corynebacterium* spp. not JK	*Fusobacterium nucleatum*	58
20	Swab from OR	Negative	*Peptostreptococcus* spp.	*Porphyromonas gingivalis*	48
27	Wound aspirate	Many segs Rare GPC	VRE	*Fusobacterium varium*	90
	Wound swab	Few segs Many RBCs No organisms	Carbapenem resistant *Klebsiella pneumoniae*	*Klebsiella pneumoniae* (*bla*_KPC-3_)	105
28	Swab from OR #1	Negative for organisms	*Enterobacter*	*Enterobacter cloacae* complex	104
	Swab from OR #2	Negative for organisms	*Enterobacter*	*Enterobacter cloacae* complex	105
		Rare segs	*E. coli*	*E. coli*	3888
29	Left hip tissue	No organisms	*E. feacalis*	*E. feacalis*	138
			*B. fragilis*	*B. fragilis*	2153
31	Right knee fluid (Swab from OR)	Rare segs No organisms	No growth	*Klebsiella pneumoniae* (*bla*_KPC-3_)	52
32	Necrotic lymph node tissue	Many segs No organisms	No growth	No detection	–
35	Right elbow fluid	Many segs No organisms	No growth	No detection	–
36	Peri-biliary	Rare GPC	Many *E. faecalis*	*Enterococcus faecalis*	181
	fluid	in clusters	Few *Klebsiella pneumoniae*	*Klebsiella pneumoniae*	35
37	Right knee femoral canal tissue	No segs No organisms	No growth	No detection	–
	Right knee tibial canal tissue	No segs No organisms	No growth	Group G Streptococcus	11
		Many segs	Many *Streptoccocus mitis/oralis*	*Finegoldia magna*	181
39	Tissue from I&D of left ankle abscess in O.R.	Some GPC in clusters	Rare MSSA	*Streptoccocus oralis*	81
				*Streptococcus infantis/peroris*	74
		Rare segs	*Staphylococcus aureus*	*Fusobacterium nucleatum*	11
41	Right 5th metatarsal bone tissue from O.R.	Rare GNB	(MSSA)	*S. aureus* (*mec*A negative)	7
				*Acinetobacter junii*	12
42	Left hip tissue	Some segs. No organisms	No growth	No detection	–
44	Left knee tissue	Some segs No organisms	No growth	*S. aureus* (*mec*A negative)	12

	Central Nervous System Specimens				
Patient	Specimen	Gram Stain	Culture results	PCR/ESI-MS	Level (GE/well)#
2	CSF #1	No organisms	No growth	*Streptococcus intermedius*	229
	CSF #2	Gram positive structures resembling cocci	No growth	*Streptococcus intermedius*	204
4	Purulent drainage from brain abscess	Many Segs Some GPC	Many *Streptoccocus mitis/oralis*	*Streptococcus intermedius*	164
7	CSF	No organisms	No growth	*Streptococcus pneumoniae/Streptococcus mitis* group	208
	Brain tissue	No organisms	No growth	*Streptococcus pneumoniae/Streptococcus mitis* group	145
12	Epidural tissue	Many segs Many GPB	Many *Propionibacterium acnes*	*Propionibacterium acnes*	184
13	CSF #1	No organisms	No growth	*Streptococcus pneumoniae/Streptococcus mitis* group	123
	CSF #2	No organisms	No growth	No detection	–
17	CSF	No organisms	No growth	No detection	–

	Cardiac tissue and Vascular specimens				
Patient	Specimen	Gram Stain	Culture results	PCR/ESI-MS	Level (GE/well)#
3	AV tissue	Few segs Rare GPC	No growth	*S. epidermidis* (*mec*A positive)	185
	Annulus tissue	Rare segs No organisms	No growth	*S. epidermidis* (*mec*A positive)	219
21	AV tissue	Negative	No growth	No detection	N/A
22	AV tissue	Few GPC Few WBC	*Abiotrophia defectiva*	*Abiotrophia defectiva*	113
	MV tissue	Few segs Few GPC	*Abiotrophia defectiva*	*Abiotrophia defectiva*	121
23	BACTEC blood culture bottle #1	N/A	No growth	No detection	–
	BACTEC blood culture bottle #2	N/A	No growth	No detection	–
34	Fluid from post-op seroma drained in O.R.	No segs No organisms	No growth	No detection	–
	Arterial Graft material extracted in O.R.	No segs No organisms	No growth	*Staphylococcus lugdunensis*	116
43	peri-Aortic valve and graft fluid	Rare segs No organisms	No growth	*Salmonella enterica*	30
	Aortic valve and graft tissue	Rare segs No organisms	No growth	*Salmonella enterica Acinetobacter junii*	122 12

# Level of Detection– Reported as genome equivalents per PCR reaction (GE/well).

* LLL  =  Left lower lobe; RLL  =  Right lower lobe; ET aspirate  =  endotracheal aspirate.

# CSF  =  cerebrospinal fluid; BAL fluid  =  bronchoalveolar lavage fluid.

Conventional culture methods were non-diagnostic in 33 of 47 cases: in 17 cases cultures were completely negative. Nine of the patients from whom respiratory specimens were collected grew either normal respiratory flora (5), or *Candida* spp. (3), or coagulase negative staphylococci (1). There was only one specimen, an endotracheal tube aspirate, from which an organism was cultured (*C. albicans*), but PCR/ESI-MS testing was negative. PCR/ESI-MS results were negative for detection in six of the 17 culture negative cases.

Bacterial pathogens were detected by PCR/ESI-MS in 60% (33/55) of the specimens in which cultures were either negative or nondiagnostic: *Streptococcus* spp. (17), *Staphylococcus aureus* (5), *Staphylococcus epidermidis* (4), *Staphylococcus lugdunensis* (1), anaerobes (4), *Salmonella enterica* (2), and *bla*_KPC-3_+ *Klebsiella pneumoniae* (1). In each case, the organism(s) detected by PCR/ESI-MS were consistent with the clinical scenario that was observed in the patient by one of our investigators (JJF). A selection of these cases requires special comment.

### Recurrent infections: (Patients 15 and 16)

Patients 15 and 16 both were on antimicrobial treatment for presumed relapses of previous *S. aureus* prosthetic knee infections. Although this suspicion was not confirmed by culture, in both cases the previously identified organism (MSSA and MRSA, respectively) was detected by PCR/ESI-MS.

### Coagulase-negative staphylococci: (Patients 3 and 10)

Coagulase negative staphylococcal infections were suspected in patients 3 and 10. Patient 3 had a history of coronary artery bypass surgery and aortic valve (AV) replacement in 1983. He was well until 2011 when he presented to an outside hospital with fever. He was diagnosed with prosthetic valve infective endocarditis based on the presence of prosthetic aortic valve vegetations and growth of methicillin susceptible *Staphylococcus epidermidis* (MSSE) in two of two sets of blood cultures. He was treated with IV vancomycin and oral rifampin for 30 days, and then transferred to our institution prior for AV replacement surgery. During surgery, Gram positive cocci (GPC) were detected on Gram stain of the valve tissue, but cultures proved to be negative. *Staphylococcus epidermidis* was detected by PCR/ESI-MS in both valve and annular myocardial tissue specimens. Given the presence of prosthetic AV vegetations, CNS in blood cultures obtained before surgery, and GPC in the valve tissue, the detection of MSSE by PCR/ESI-MS is noteworthy and bears significance.

In contrast, the growth of methicillin resistant CNS in the synovial fluid cultures from right knee fluid in patient 10, obtained 10 days prior to initiation of antibiotics, and subsequent negative aerobic intra-operative cultures and growth of only one colony of CNS in one of three anaerobic cultures obtained from during extraction of the infected prosthesis was of uncertain significance. Consideration of PCR/ESI-MS results eliminates any doubt, as MRSE was identified in all three specimens.

### Mixed aerobic and anaerobic infections: (Patients 18, 20, 27, 33, 39, 40 and 41)

PCR/ESI-MS detected anaerobic organisms that were missed by culture in eight cases. *Porphyromonas gingivalis* was not appreciated in the culture of neck abscess fluid from patient 20, which was culture negative. And *Bifidobacterium dentium* was detected in BAL fluid from patient 33 that was only notable for *Candida albicans* in culture. *Rothia mucilaginosa* as well as *C. albicans* were identified in the respiratory specimens from patient 40, whose cultures were non-diagnostic (*i*.*e*., normal flora). Culture and PCR/ESI-MS also disagreed on patient 27: VRE was cultured from the original wound culture, but PCR/ESI-MS detected *Fusobacterium varium*.

PCR/ESI-MS performed particularly well with polymicrobic infections that included both aerobic and anaerobic pathogens. *S. intermedius* and MSSA grew in both aerobic and anaerobic cultures, from patients 18 and 41, respectively, but no strictly anaerobic organisms were cultured. *Fusobacterium necrophorum* and *Streptococcus* spp. were detected in purulent liver abscess drainage from patient 18, and MSSA and *F. necrophorum* were identified in the infected metatarsal bone from patient 41. Patient 38 had MSSA and *C. albicans* detected by culture and PCR/ESI-MS in the pleural fluid sample, but *Bilophila wadsworthia* was only detected by PCR/ESI-MS, and not in the anaerobic culture. Likewise, *Streptoccocus oralis* was found in ankle abscess tissue by both aerobic culture and PCR/ESI-MS from patient 39, but only PCR/ESI-MS detected *Finegoldia magna*.

### Streptococcal infections: (Patients 1, 2, 4, 5, 7, 8, 13, 14, 18, 30, 37, 39 and 46)

Of the 13 patients (17 samples) from whom *Streptococcus* spp. were detected by PCR/ESI-MS, the only specimens from which *Streptococcus* was recovered by culture were from patients with brief or no antibiotic treatment prior to specimen collection. Two specimens that grew *Streptococcus mitis*/*oralis*: 1) purulent brain abscess drainage from Patient 4 obtained after two days of antibiotic treatment, and 2) the ankle abscess tissue from patient 39– three days after antibiotics were discontinued. *S. intermedius* was cultured from purulent liver abscess fluid from patient 18 after one day of antibiotic treatment. In patients who had received more than two days of antibiotic treatment, streptococci were no longer cultured. And, in the case of patient 14, one day of treatment was sufficient to suppress growth of streptococci: Left knee synovial fluid cultures from patient 14 grew α-hemolytic streptococci prior to initiation of antibiotics, but after one day of antibiotics, when the knee was drained in the OR, all cultures were negative. PCR/ESI-MS detected viridans streptococci/*S. pneumoniae/mitis* group in three of three surgical specimens from patient 14.

PCR/ESI-MS appeared to offer a particular advantage in detection of pneumococci from both cerebrospinal fluid (CSF), and bronchoalveolar lavage (BAL) fluid. Lumbar puncture (LP) was delayed in two cases of pneumococcal bacteremia and sepsis with presumed meningitis (Patients 7 and 13). In both cases, CSF cultures were negative, but *S. pneumoniae* were detected by PCR/ESI-MS (thereby “salvaging” clinical decision making). Dexamethasone was not administered in either case. Interestingly, PCR/ESI-MS did not remain positive in the CSF indefinitely. CSF from a follow-up LP two weeks later on patient 13 was negative by both culture and PCR/ESI-MS (see table 2). In two cases of apparent pneumococcal pneumonia (Patients 5 and 8) and a third case of viridans streptococcus pneumonia (patient 46), culture was either unable to detect *S. pneumoniae*, or unable to distinguish pathogenic streptococci from normal respiratory flora.

### Prosthetic arterial graft infections: (Patients 33 and 43)

Infection of prosthetic intravascular graft material is a difficult problem, as vascular grafts are not readily exchanged. Endovascular graft infection was suspected in Patients 33 and 43. Both patients were bacteremic, and both were on antimicrobial treatment prior to surgical extraction of the vascular grafts. Cultures of graft material and surrounding tissue were negative, but in both cases, the organism that had grown in the initial blood cultures was detected by PCR/ESI-MS from the extracted the graft material.

### Carbapenem resistant*Enterobacteriaceae* (CRE): (Patients 27 and 31)

CRE infections were detected in one patient by culture and two patients by PCR/ESI-MS. In patient 27, *K. pneumoniae* that tested positive for KPC-3 by PCR for the *bla*_KPC_ gene was recovered from an infected surgical wound. PCR/ESI-MS detected both the pathogen (*K. pneumoniae*), and the resistance gene (*bla*_KPC-3_). Patient 31 was known to be colonized with *bla*_KPC-3_+ *Klebsiella pneumoniae*, and although cultures of synovial fluid from her right knee were negative, PCR/ESI-MS detected *K. pneumoniae* (also positive for *bla*_KPC-3_).

### Extended antibiotic treatment and serial specimens: (Patients 13 and 32)

Just as with culture, duration of antibiotic treatment does influence ability of PCR/ESI-MS to detect evidence of a pathogen. In the case of serial CSF samples from patient 13, a patient with *S. pneumoniae* bacteremia and sepsis, *S. pneumoniae* was only detected by PCR/ESI-MS in the first CSF sample. In the case of patient 32, both culture and PCR/ESI-MS of neck abscess tissue were negative after more than 34 days of empiric antibiotic treatment.

### *Acinetobacter junii*: (Patients 6, 16, 41, 43)

In these four cases *Acinetobacter junii* was detected by PCR/ESI-MS in tissue and synovial fluid specimens collected during surgical resection and drainage of infected tissue. *Acinetobacter junii* is unlikely to represent a pathogen in these cases. This organism appears to have been detected as an artifact of the tissue extraction process.

## Discussion

PCR/ESI-MS is an emerging diagnostic technology that is capable of rapid detection of microorganisms directly from clinical specimens. As this PCR-based approach requires only the presence of small amounts of DNA for amplification, bacteria that have been “killed” by bactericidal antibiotics (*e*.*g*., β-lactams, aminoglycosides or quinolones) or are in stationary phase from the effect of bacteriostatic drugs (*e*.*g*., linezolid, macrolides) can be detected if sufficient DNA for amplification is present in the sample. Up to this time, data testing this assertion in the clinical arena have not yet been provided.

In our series, 72% (55/76) of cultures obtained following initiation of antimicrobial treatment were nondiagnostic. In contrast, PCR/ESR-MS detected organisms in 83% (39/47) of cases and 76% (58/76) of the specimens. Calculation of the Kappa coefficient confirmed poor agreement between conventional cultures and PCR/ESI-MS. The poor agreement is primarily attributable to detection of bacteria by PCR/ESI-MS in culture negative specimens. PCR/ESI-MS detected ≥ one bacterial pathogen(s) in 60% (27/45) of the culture negative specimens. In 67% of negative culture cases (18/27), an organism that was consistent with the clinical scenario was detected by PCR/ESI-MS.

Reliance on clinical judgment to distinguish colonizers and contaminants from true pathogens is required with any microbiology test result. As with culture-based identification of organisms from clinical samples, the correct interpretation of PCR/ESI-MS results requires an appreciation for the clinical context associated with the specimen tested. In several cases the organisms detected by PCR/ESI-MS were consistent with contaminants that would have been unlikely to alter patient management. For example, detection of *Candida* spp. in respiratory secretions by either culture or molecular methods does not merit treatment in our relatively immunocompetent patient population. But, as evidenced in the case of Patient 6, the role of other potential pathogens still needs to be defined. In this case, low level detection (17 genome copies/well) of *Propionibacterium acnes*, a pathogen with well described association with prosthetic shoulder joint infections, in culture negative right shoulder synovial fluid would pose a challenge for the clinician responsible for interpreting this additional data.

Selecting appropriate antimicrobial therapy for patients with evidence of infection, but negative cultures is a common dilemma in practice. The implications of our findings are profound: that antimicrobial treatment in “culture negative” cases can be directed against both pathogens and genetic markers of resistance (*i*.*e*., *mec*A in MRSA, mutations in *qrdr*, *bla*_KPC_, etc.) that are readily identified by PCR/ESI-MS. Particularly compelling supporting evidence for pathogen detection derives from our two cases of breakthrough recurrent prosthetic knee infections that occurred while the patients were taking chronic suppressive antibiotics (patients 15 and 16): the previous organism was not recovered in tissue cultures taken during extraction of the infected knee prostheses in either case, but was detected by PCR/ESI-MS. As well as our case of culture and Gram stain positive streptococcal septic arthritis (Patient 14), in which a single preoperative dose of cefazolin was sufficient to cause surgical cultures to be negative. The disappearance of PCR evidence of *S. pneumoniae* in a second CSF specimen from patient 13, who was recovering from pneumococcal bacteremia and sepsis, suggests that organism detection may be eradicated with effective antimicrobial treatment. This finding may help with decisions to tailor and/or stop therapy. These early findings could have impact on the current status of “duration of therapy” and antibiotic stewardship.

Limitations of this study include: *i*) the small sample size and lack of a control group; *ii*) relevance of “mixed cultures”; and *iii*) co-identification of streptococci (viridians streptococci, *Streptococcus mitis* and pneumococcus). This study was performed as an open investigation, and not a validation; and is not appropriate for, nor was it designed to calibrate sensitivity or specificity. Our PCR primers successfully captured organisms not detected by culture, but we maintain that microbiological culture results are still the “gold standard” for comparison. Based upon studies such as this, that “standard” cannot be applied as the evidence it offers is not present, but combining broad range PCR with mass spectrometry or 16S ribosomal gene sequencing has appeal for selected situations in the clinical microbiology lab. In addition, the role of fungal, viral or parasitic infections was also not evaluated in this small series. Truly, larger studies are needed.

The everyday practice of treating patients with empiric antibiotic regimes provides an enormous opportunity for novel approaches to target antimicrobial therapy and “salvage” both individual treatment regimens as well as institutional antimicrobial stewardship efforts. These results suggest that PCR/ESI-MS may have a role in detection of clinically relevant pathogens from specimens obtained following initiation of antimicrobial treatment when cultures are negative. Larger studies are planned to determine if PCR/ESI-MS can assist in the clinical evaluation and treatment of patients on empiric antimicrobial treatment for suspected infection with negative cultures.

## References

[1] Bhatia NS, Farrell JJ, Sampath R, Ranken R, Rounds MA, et al (2012) Identification of *Streptococcus intermedius* central nervous system infection by use of PCR and electrospray ionization mass spectrometry. J Clin Microbiol 50: 4160–62. 23035188 10.1128/JCM.01296-12 PMC3502992

[2] Puskarich MA, Trzeciak S, Shapiro NI, Arnold RC, Horton JM, et al (2011) Association between timing of antibiotic administration and mortality from septic shock in patients treated with a quantitative resuscitation protocol. Crit Care Med 39: 2066–71. 21572327 10.1097/CCM.0b013e31821e87ab PMC3158284

[3] Houck PM, Bratzler DW, Niederman M, Bartlett JG (2002) Pneumonia treatment process and quality. Arch Intern Med 162: 843–4. 10.1001/archinte.162.7.843-a 11926866

[4] McGarvey RN, Harper JJ (1993) Pneumonia mortality reduction and quality improvement in a community hospital. QRB Qual Rev Bull 19: 124–30. 8493027 10.1016/s0097-5990(16)30605-4

[5] Fitch MT, van de Beek D (2007) Emergency diagnosis and treatment of adult meningitis. Lancet Infect Dis 7: 191–200. 17317600 10.1016/S1473-3099(07)70050-6

[6] Aronin SI, Peduzzi P, Quagliarello VJ (1998) Community-acquired bacterial meningitis: risk stratification for adverse clinical outcome and effect of antibiotic timing. Ann Intern Med 129: 862–9. 9867727 10.7326/0003-4819-129-11_part_1-199812010-00004

[7] Proulx N, Frechette D, Toye B, Chan J, Kravcik S (2005) Delays in the administration of antibiotics are associated with mortality from adult acute bacterial meningitis. QJM 98: 291–8. 15760921 10.1093/qjmed/hci047

[8] Sampath R, Hall TA, Massire C, Li F, Blyn LB, et al (2007) Rapid Identification of Emerging Infectious Agents Using PCR and electrospray ionization mass spectrometry. Ann NY Acad Sci 1102: 109–20. 17470915 10.1196/annals.1408.008 PMC7167958

[9] Ecker DJ, Sampath R, Li H, Massire C, Matthews HE, et al (2010) New technology for rapid molecular diagnosis of bloodstream infections. Expert Rev Mol Diagn 10: 399–415. 20465496 10.1586/erm.10.24

[10] Caliendo AM (2011) Multiplex PCR and emerging technologies for the detection of respiratory pathogens. Clin Infect Dis 52: S326–30. 21460291 10.1093/cid/cir047 PMC7107927

[11] Kaleta EJ, Clark AE, Johnson DR, Gamage DC, Wysocki VH, et al (2011) Use of PCR coupled with electrospray ionization mass spectrometry for rapid identification of bacterial and yeast bloodstream pathogens from blood culture bottles. J Clin Microbiol 49: 345–53. 21048006 10.1128/JCM.00936-10 PMC3020459

